# Spontaneous Aortocaval Fistula Due to Ruptured Infrarenal Aortic Aneurysm

**Published:** 2012-10-30

**Authors:** A Afshar far, A Amir Derakhshanfar, Kh Atqiaee, S Lotfolah zadeh, M R Sobhiyeh, S Jabbehdari

**Affiliations:** 1 Professor of General and Vascular Surgery, Shahid Beheshti University of Medical Sciences and Health Services, Shohadaye Tajrish Hospital, Tehran, Iran; 2 General and Vascular Surgen, Shahid Beheshti University of Medical Sciences and Health Services, Shohadaye Tajrish Hospital, Tehran, Iran; 3 Resident of General Surgery, Shahid Beheshti University of Medical Sciences and Health Services, Shohadaye Tajrish Hospital, Tehran, Iran; 4 Students' Research Committee, Faculty of Medicine, Shahid Beheshti University of Medical Sciences, Tehran, Iran

**Keywords:** Aortocaval Fistula, Aortic Aneurysm

Dear editor,

Aortocaval Fistula (ACF) is a rare condition of an Abdominal Aorta Aneurysm (AAA). ACF caused by perforation of atherosclerotic infrarenal aortic aneurysm into the adjacent IVC, iliac vein, or left renal vein.(1) Its incidence is approximately 1-2% which increases to 2-6.97% in the presence of ruptured AAA.(2) ACF is reported more in males (98%) with an average age of 64 years.(3) The common presentations of ACF includes a palpable abdominal mass, continuous bruit or thrill.(2) The early Diagnosis of ACF is very crucial due to the high mortality rate of approximately 30% .(4)

A 63-year-old man presented to Emergency Department with sudden left flank pain.

Abdominal physical examination revealed palpable mass, tenderness and self-guarding in left flank area without rebound tenderness. All peripheral pulses were present.Sonography of abdomen displayed infrarenal AAA with 63mm diameter. CT-images demonstrated infrarenal aortic aneurysm with fistula to IVC arising from aneurysm lumen that penetrated to retroaortic renal vein situated beneath the aneurysm (Figure).

**Figure 1:**
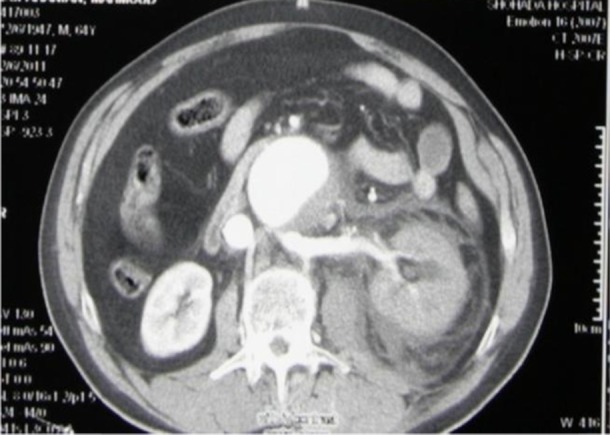
Abdominal CT of the patient showing large infrarenal aortic aneurism and fistula to IVC

At laparotomy No hematoma or active bleeding existed in peritoneal space. Followed by controlling blood flow of aneurysm neck, distal common iliac arteries were clamped. Therefore, renal veins became noticeable posterior the aorta. IVC was dilated excessively 4 cm and trill was palpable on aneurysmal site. Further investigations revealed that exact site of fistula at the entrance of left renal vein to IVC.IVC was repaired and renal vein was ligated and aneurysm repaired using 20 tubular Dacron graft. There are several causes for ACF including spontaneous rupture of atherosclerotic aneurysm directly into adjacent IVC,(5) penetrating abdominal trauma,(6) iatrogenic trauma at the lumbar disc surgery,(7) mycotic aneurysm, and connective tissue disorders.(5) 80% of ACF cases are due to rupture of an aortic aneurysm.(8) 

Early diagnosis of ACF prior to surgery is vital and also difficult due to rarity of complication and non-specificity of the signs and symptoms. Standard signs of ACF as a result of aneurysmal disease, include acute abdominal pain, hypotension, and pulsatile abdominal mass.(9) However standard signs were only represented by 50% of the patients.(10) 

Specific treatment for ACF is operative transaortic closure of fistula and placement of prosthetic graft or endovascular repair of fistula.(8)
